# Roll-to-Roll SiOx Synthesis on Polyethylene Terephthalate Film by Atmospheric-Pressure Plasma-Assisted Chemical Vapor Deposition

**DOI:** 10.3390/ma17194694

**Published:** 2024-09-24

**Authors:** Yukihiro Kusano, Kim Bredgaard, Huifang Pan, Alexander Leo Bardenstein

**Affiliations:** 1 Danish Technological Institute, 2630 Taastrup, Denmark; alb@teknologisk.dk; 2 Department of Marine Resources and Energy, Tokyo University of Marine Science and Technology, Shinagawa, Tokyo 108-8477, Japan; 3 Vetaphone ApS, 6000 Kolding, Denmark

**Keywords:** silicon oxide, atmospheric-pressure plasma-assisted chemical vapor deposition, roll-to-roll, polyethylene terephthalate, X-ray photoelectron spectroscopy, Fourier transform infrared spectroscopy

## Abstract

Silicon oxide (SiOx) coatings are attracting significant attention and are widely used in industrial applications. They can be prepared by plasma-assisted chemical vapor deposition (PACVD). PACVD at atmospheric pressure (AP-PACVD) is often employed to synthesize SiOx coatings, but it has generally not been scaled up to an industrially viable level. In the present work, a SiOx coating was continuously deposited onto a polyethylene terephthalate film using industrial-scale roll-to-roll type AP-PACVD. 1,1,3,3-Tetramethyldisiloxane (TMDSO) and tetraethoxysilane (TEOS) were selected as precursors. The elemental compositions and chemical structures of the SiOx coatings were characterized, and oxygen and water-vapor transmission rates were measured. The SiOx coating using TEOS exhibited better barrier properties than that using TMDSO, corresponding to the high oxygen content, high SiO_2_ content, and high siloxane network content in the SiOx coating.

## 1. Introduction

Silicon oxide (SiOx) coatings are used in a variety of industrial applications, including dielectric and insulating coatings in semiconductor [1] and battery applications [2], hard tribological coatings in mechanical applications [3], transparent coatings in optical applications [4], and gas barrier coatings in food packaging applications [5]. They are usually prepared via the thermal oxidation of silicon [6], sol–gel methods [7], and plasma-assisted chemical vapor deposition (PACVD) [8]. When SiOx coatings are deposited onto organic materials such as plastic films, PACVD is preferably employed to avoid thermal damage to the materials. A gaseous organosilicon compound is fed into plasma as a precursor, together with O_2_ gas as appropriate, which is decomposed and oxidized to form SiOx films, which generally contain not only silicon and oxygen atoms but also carbon and hydrogen atoms. SiOx coatings with a high carbon content typically exhibit a polymer-like soft and flexible nature, while those with a low carbon content usually exhibit high-density fragile properties. When SiOx coatings are synthesized onto plastic films for improving gas barrier properties, a good balance is required between flexibility, to avoid the cracking of the coating, and high density, to achieve improved gas barrier properties.

The organosilicon compounds commonly used as CVD precursors include hexamethyldisiloxane (HMDSO) [9,10,11,12,13,14,15,16,17], 1,1,3,3-tetramethyldisiloxane (TMDSO) [9,18], tetraethoxysilane (TEOS) [17,19,20,21,22,23], tetramethoxysilane (TMOS) [24], trimethylsilane (TMS) [25,26,27], hexamethylcyclotrisiloxane (HMCTS) [28], 2,4,6,8-tetramethylcyclotetrasiloxane (TMCTS) [23,29], octamethylcyclotetrasiloxane (OMCTS) [23,29,30], polyhydrogenmethylsiloxane [23], tetramethyldisiloxane [23], 3-aminopropyl-triethoxysilane (APTES) [31], and polydimethylsiloxane (PDMS) [32]. HMDSO [9,10,11,12,13,14,15,16,17] and TEOS [9,18] are the most widely used precursors due to their moderate prices, relatively low flammability, and low toxicity. TMOS [24] has a molecular structure similar to TEOS. However, TEOS is more preferably used since the hydrolysis of TEOS produces ethanol, which is less toxic than methanol produced by the hydrolysis of TMOS. TMS [25,26,27] is highly flammable, and thus special care is needed during its handling. Cyclic precursors have also been tested, including HMCTS [28], TMCTS [23,29], and OMCTS [23,29,30]. They are easily polymerized by ring-opening reactions. However, they do not necessarily result in the synthesis of dense crosslinked SiOx films. Since OMCTS has four dimethylsiloxane units, it is called D4, which is considered to be toxic [33] and strictly regulated in the EU. Therefore, it is advisable to avoid the use of D4 or the risk of synthesizing it in PACVD. It is noted that there is a potential risk that unreacted precursors may remain in the coating or the plastic film or that the precursor may be modified in the plasma and synthesize unwanted substances. These precursors are usually liquid at room temperature, but solid precursors such as HMCTS can also be used as long as they are volatile substances and their sublimation pressure is high enough. However, one should be aware that HMCTS is a cyclic precursor and has a risk of synthesizing OMCTS (D4).

When the vapor pressure of the organosilicon precursor selected is high enough, the precursor can be directly introduced to the plasma using a gas flow controller. However, a bubbling device or similar means is often used to ensure the introduction of a sufficient flow of the precursor into the plasma. For the carrier gas of the bubbling device, an inert gas, such as helium, argon, or N_2_ gas, or a reactive gas, such as O_2_ gas, is used. It is reported that PDMS [32] can be used as a precursor. However, since it is not a volatile liquid, and its vapor pressure is almost zero, the method to introduce a PDMS vapor into the plasma must be established to achieve gas-phase reactions in the CVD process. PACVD is conventionally employed at low gas pressures. It is argued that sufficient ion bombardment in a low-pressure plasma is preferred as this rearranges the atomic displacement, reducing defects and forming high-density crystallites [34] that exhibit high barrier performances. However, it is reported that SiOx materials can also be synthesized by PACVD at atmospheric pressure (AP-PACVD) [9,10,11,12,13,14,15,16,17,18,19,20,21,22,23,25,26,27,28,29,30,31,32,33]. Since high gas barrier performances of SiOx coatings are reported when using AP-PACVD, it can be said that efficient ion bombardment is not of primary importance for achieving gas barrier properties. The advantage of atmospheric-pressure plasma processing is that expensive vacuum equipment is not needed, and the processing of large objects and continuous treatments can be realized [35]. In addition, since the plasma is generally sustained only around the electrodes where the CVD coatings are mostly synthesized, the maintenance of the plasma unit is much simpler than that of low-pressure PACVD. For the plasma source of AP-PACVD, a dielectric barrier discharge (DBD) and a cold plasma torch are usually used. DBD is the most popular atmospheric-pressure plasma in materials processing [35,36,37,38]. DBD is driven by an alternating current (AC) voltage and generated between electrodes. At least one dielectric insulator is placed between the electrodes to block the direct current. The gap between the electrodes is limited to a few millimeters to sustain a stable plasma. If the substance to be coated with AP-PACVD SiOx coatings is thin enough, DBD is preferred since the substance can be directly exposed to the DBD. Plasma torches are thermal plasmas applied for thermal processing, including the plasma pyrolysis used for recycling [38]. Meanwhile, a non-thermal atmospheric-pressure plasma jet called a cold plasma torch has been developed [19,39,40]. In a cold plasma torch, plasma is generated and extended to an open environment by a high-speed gas flow. Many of these cold plasma torches are based on the DBD configuration. Cold plasma torches need to use noble gases such as helium and argon as carrier gas to extend the plasma, resulting in expensive operation costs. It is noted that the plasma generated at the electrodes is much more reactive than the plasma extending into an open environment. Therefore, a cold plasma torch is typically less energy-efficient than a DBD. Microwave power can also be used to generate plasma, which is extended by a high-speed gas flow. The plasma properties of the atmospheric-pressure microwave plasma jets are similar to those of the atmospheric-pressure gliding arcs [41]. However, SiOx synthesis using gliding arcs has not been reported. An outstanding advantage of employing atmospheric-pressure plasmas is that an air-to-air, roll-to-roll process can be achieved [21,32,42,43,44]. However, the demonstration of industrially feasible large-scale AP-PACVD has not been reported for the synthesis of SiOx coatings in the literature.

SiOx materials are characterized in terms of their elemental compositions, chemical and functional structures, morphology, observation of defects and cracks, mechanical properties, electrical and optical properties, and gas barrier properties. X-ray photoelectron spectroscopy (XPS) is used for characterizing the elemental composition and chemical structures of SiOx coatings. Characterization of regional silicon Si2p spectra gives direct information on the stoichiometry of SiOx [45], while measurement of carbon C1s spectra provides the bonding states of the remaining carbon atoms in the SiOx coatings. Fourier transform infrared (FTIR) analysis is a useful technique to characterize the chemical structure of the SiOx coatings. However, the interpretation of the FTIR spectra is not straightforward since there are discrepancies in the identification of molecular structures in the literature. For investigating gas barrier properties, water-vapor and oxygen transmission rates (WVTR and OTR) are commonly measured.

In the present work, a large-scale roll-to-roll type DBD unit is operated to synthesize SiOx coatings on a polyethylene terephthalate (PET) film. TMDSO or TEOS is used as a precursor. Here, PET is favorably used for beverages and food packaging applications due to its recyclability and physical properties, such as inertness and low diffusivity [46]. Surface wetting properties, elemental compositions, and chemical structures are characterized using contact angle measurements, XPS analysis, and FTIR. WVTR and OTR of the coated PET films are measured.

## 2. Materials and Methods

### 2.1. Sample Preparation

**Figure 1:**
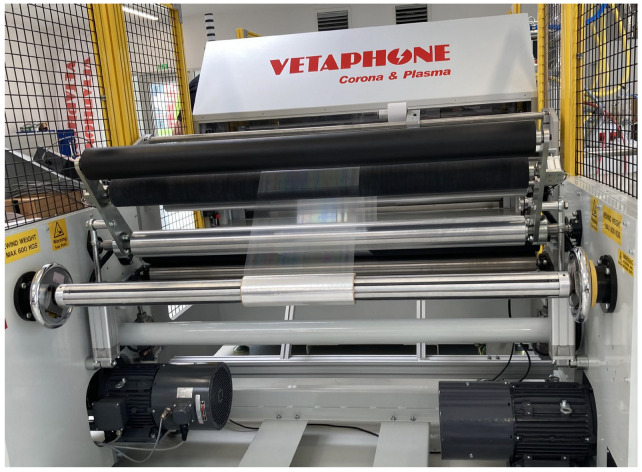
A photo image of the roll-to-roll AP-PACVD system for synthesis of SiOx coating on a PET film.

**Figure 2:**
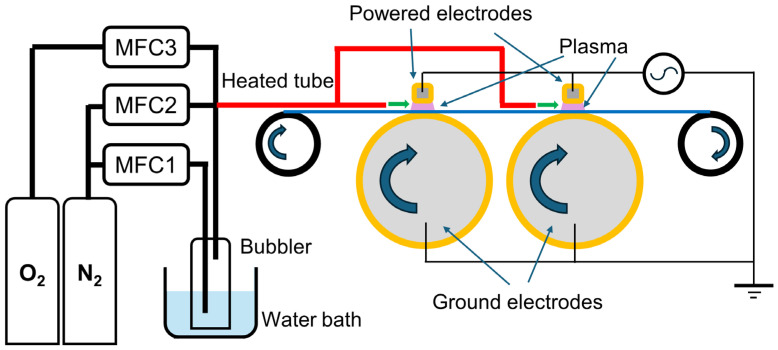
A schematic diagram of the roll-to-roll AP-PACVD system, including a gas feeding system. MFC: mass flow controller.

A roll-to-roll type DBD unit was used to synthesize SiOx coatings. A photo image and a schematic diagram of the plasma unit are shown in Figure 1 and Figure 2, respectively. A PET film (vPET, 23 μm thick, Folia PET corona 23 my/333 mm, or PET, 150 μm thick, Folia CASTFOL APET-150-NN-00-000 333 mm, EUROCAST Sp. Z o.o., Strzebielino, Poland) was introduced to a plasma zone so that the SiOx coating could be deposited onto it. The coated PET film was subsequently wound up. Although the plasma unit was capable of processing up to 1,300 mm-wide films, in the present work, 333 mm-wide PET films were used to simplify the handling of the specimens. AP-PACVD was carried out using two DBD cassettes. Each cassette consisted of water-cooled powered electrodes aligned perpendicularly to the transverse direction of the PET film. They were covered with quadrangular prism-shaped alumina ceramics that served as dielectrics. Cylindrical ground electrodes were also water-cooled and covered with alumina.

A liquid precursor was stored in a bubbler through which a nitrogen carrier gas was fed. The precursor-containing nitrogen gas and oxygen gas were mixed alongside an extra nitrogen gas as a dilute gas when appropriate. As a precursor, TMDSO or TEOS was used (Merck KGaA, Darmstadt, Germany). The bubbler was placed in a water bath to control the temperature of the precursor. TMDSO was handled at room temperature. The bubbler of TEOS was heated by the water bath at 60 °C. When TEOS was used as a precursor, the tubing connected to the plasma unit was heated at 70 °C by a resistive heater to avoid condensation of TEOS. The gas mixture was fed to each DBD cassette.

The DBD was driven by an AC power supply (Vetaphone ApS, Kolding, Denmark) at a frequency of approximately 40 kHz. The power consumed by the DBD was directly measured. The conditions selected are listed in Table 1. The flowrates of TMDSO and TEOS were estimated using the database of saturated vapor pressures under the assumption that the vapor pressure of each precursor was saturated in the bubbler. The vapor pressure of TMDSO at 25 °C is 1.80 × 10^4^ Pa [47]. The vapor pressure *P*_TEOS_ [Pa] of TEOS is expressed as a function of temperature *T* [K] [48].

(1) $$P_{TEOS}[Pa]=10^{9.17312-\frac{1,561.277}{T-67.572}}$$

The estimated flowrates of TMDSO and TEOS for the flowrates of the carrier gas were 0.50 standard liter per minute (SLM) and 0.39 SLM, respectively.

### 2.2. Characterization Methods

Optical transmission of the coated and uncoated PET films was measured using UV/Vis/NIR spectroscopy in a range between 300 and 850 nm with a scan sped of 120 nm min^−1^ and a resolution of 1 nm (Lambda 18, PERKIN ELMER, Rodgau, Germany). Mean optical transmission rates were calculated between 400 and 850 nm.

Static contact angles of deionized water and diiodomethane were measured on the PET films before and after the SiOx deposition in air at room temperature. Here, deionized water is characterized by its significantly polar nature and is commonly used for contact angle measurement [49]. Diiodomethane is also often used as a test liquid, exhibiting a non-polar nature. The values of the polar and dispersion components of the surface tension of deionized water are 51 mJ m^–2^ and 21.8 mJ m^–2^, respectively, while those of diiodomethane are 0 mJ m^–2^ and 50.8 mJ m^–2^, respectively. Subsequently, the polar and dispersion components of the surface tension of the SiOx coatings and the PET film were calculated [50,51,52].

XPS data were collected using a monochromatic Al Kα X-ray source with a lateral resolution of 30 mm (K-alpha; Thermo Fisher Scientific, Oxford, UK) to study the elemental composition at the PET film surfaces. Atomic concentrations of each element were calculated by determining the relevant integral peak intensities with linear background subtraction. Regional analyses of C1s and Si2p spectra were carried out. The full-width at half-maximum of the peaks for C1s and Si2p was fixed at 1.5 eV for curve fitting.

Attenuated total reflection FTIR (ATR-FTIR) spectroscopy was employed (diamond prism, NICOLET iS50 FT-IR, Thermo Fisher Scientific, Waltham, MA, USA) with 32 scans and a resolution of 4 cm^–1^. The FTIR spectrum of the uncoated PET film was subtracted from the FTIR spectra of the SiOx-coated PET film to characterize the chemical structures of the SiOx coatings.

OTR was determined in accordance with ASTM D 3985 at 23 °C with 0% relative humidity (RH) (Mocon, OX-TRAN 2/22—Eti no. 43. AMETEK MOCON, Brooklyn Park, MN, USA). WVTR was determined in accordance with ASTM 1249 at 38 °C with 90% RH (Mocon, PERMATRAN-W 3/34—Eti no. 45, AMETEK MOCON, Brooklyn Park, MN, USA).

## 3. Results and Discussion

### 3.1. Appearance of Coating

**Figure 3:**
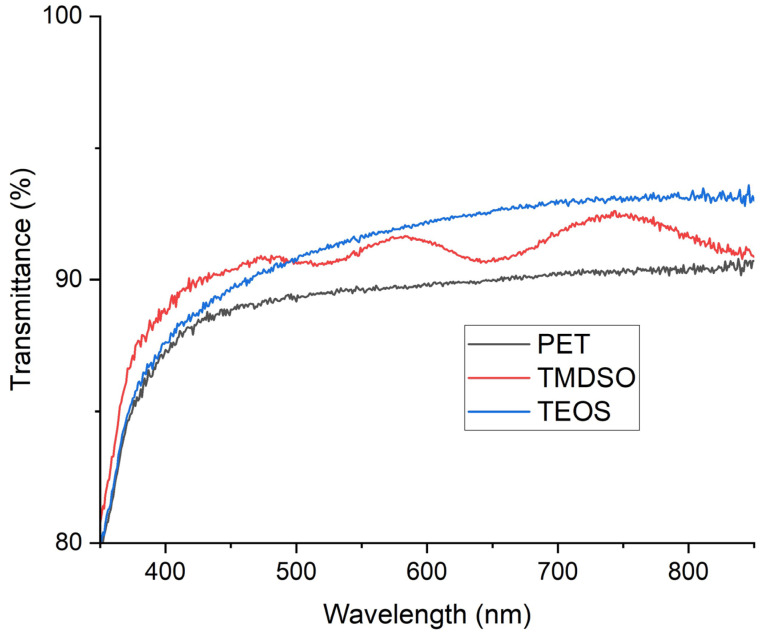
Optical transmission of the SiOx-coated and uncoated PET films.

The SiOx coatings were fairly uniformly deposited onto the PET film. The thickness of the coatings was estimated to be approximately 200 and 100 nm for TMDSO and TEOS, respectively. Figure 3 illustrates the optical transmission of the coated and uncoated PET films. The mean optical transmission rate of the uncoated PET was 89.7%. Those of the SiOx-coated PET films using TMDSO and TEOS were 91.2 and 91.8%, respectively, indicating that the synthesized SiOx films were significantly transparent in the visible optical range. The refractive index of PET is usually in a range between 1.5 and 1.7 [53]. On the other hand, the reported refractive index of the SiOx coating is between 1.5 and 1.9 and decreases as the oxygen content in the coating increases [54]. The oxygen contents of the SiOx in the present work were high, as shown in Section 3.3, and the refractive index is seen to dip below 1.6. It is indicated that the difference in the transmittance of the uncoated and coated PET is due to lower refractive indexes of the SiOx coatings than that of the PET. The optical transmittance of the SiOx coating using TMDSO showed a detectable dependence on the wavelength since the thickness was large enough.

### 3.2. Wetting Characteristics

**Figure 4:**
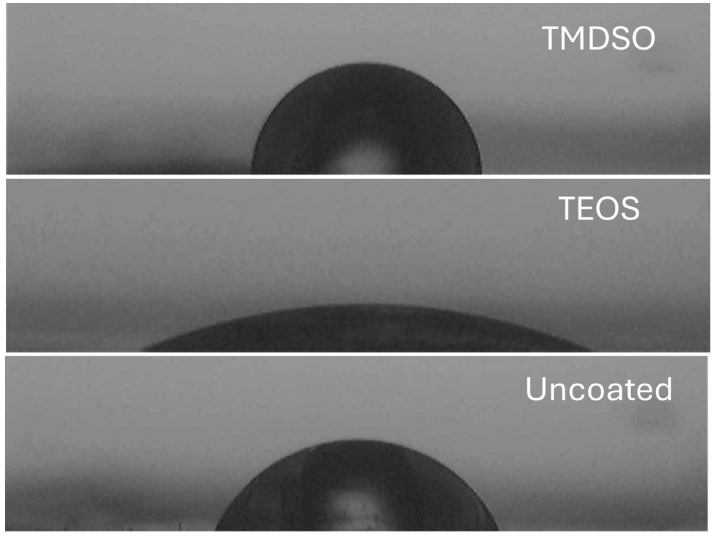
Photo images of water droplets on the SiOx coatings and the uncoated PET.

The measured contact angles and calculated surface-tension values of the SiOx coatings are summarized in Table 2. Figure 4 exemplifies photo images of water droplets on the coated and uncoated surfaces. The SiOx coating using TMDSO showed lower contact angles of deionized water and diiodomethane than the uncoated PET film, and thus, it is concluded that both the polar and dispersion components of the surface tension were lowered by the SiOx deposition using TMDSO. On the other hand, the SiOx coating using TEOS exhibited a significantly lowered contact angle of water and an increased contact angle of diiodomethane.

### 3.3. XPS Analysis

**Figure 5:**
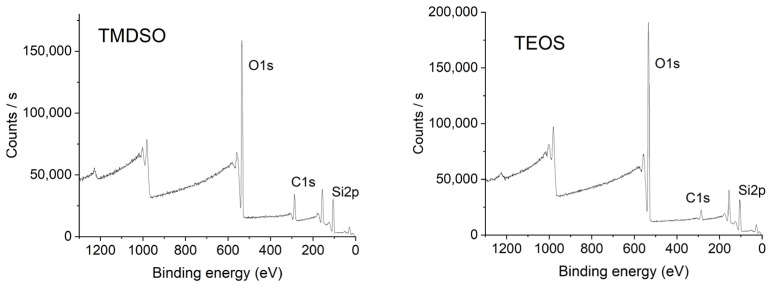
XPS survey spectra of SiOx coatings using TMDSO and TEOS.

**Figure 6:**
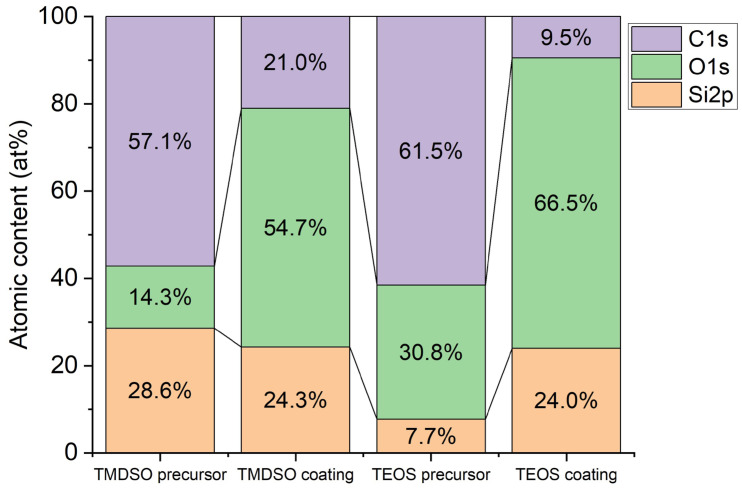
Comparison of atomic compositions of TMDSO precursor, TMDSO coating, TEOS precursor, and TEOS coating.

XPS analysis was carried out to measure the elemental composition of the SiOx coatings using TMDSO and TEOS. The result is shown in Figure 5 and summarized in Table 3. It is further compared with the elemental compositions of the precursors in Figure 6, calculated from the molecular formulae of TMDSO and TEOS. The O/Si atomic ratios of the TMDSO and TEOS coatings were higher than the stoichiometric rate of SiO_2_, indicating that some of the oxygen atoms bond with carbon atoms. The O/Si ratio of the TEOS precursor is 4, and thus, a certain number of Si-O bonds must be cut during the CVD process.

**Figure 7:**
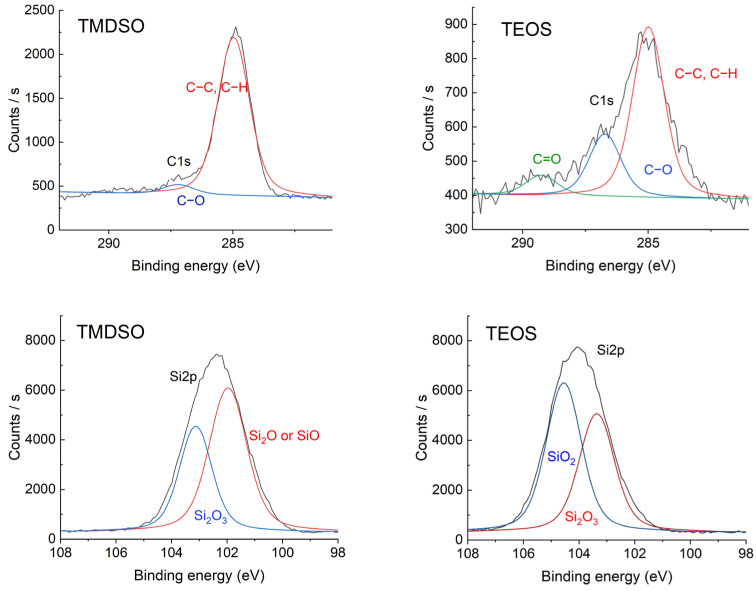
Regional C1s and Si2p XPS spectra of SiOx coatings using TMDSO and TEOS.

Regional C1s and Si2p XPS were measured. The results are shown in Figure 7 and summarized in Table 4. The result of the SiOx coating using TMDSO indicates that most carbon atoms were not oxidized. This is the reason the SiOx coating using TMDSO was rather hydrophobic (Table 2). The Si2p binding energy gives an idea about the oxidation level of silicon atoms [45]. It is considered that the 3-dimensional network siloxane structure corresponding to the stoichiometric SiO_2_ is desirable to realize high-density SiOx coatings so that high gas barrier properties can be expected. The SiOx coating using TMDSO does not reveal the SiO_2_ structure while demonstrating low-level oxidation such as Si_2_O, SiO, and Si_2_O_3_. It is noted, however, that the differences in binding energy are approximately 1 eV and that the measured data can be affected by slight charging of specimen surfaces. Therefore, the identification above is not a definitive assignment.

### 3.4. FTIR Analysis

**Figure 8:**
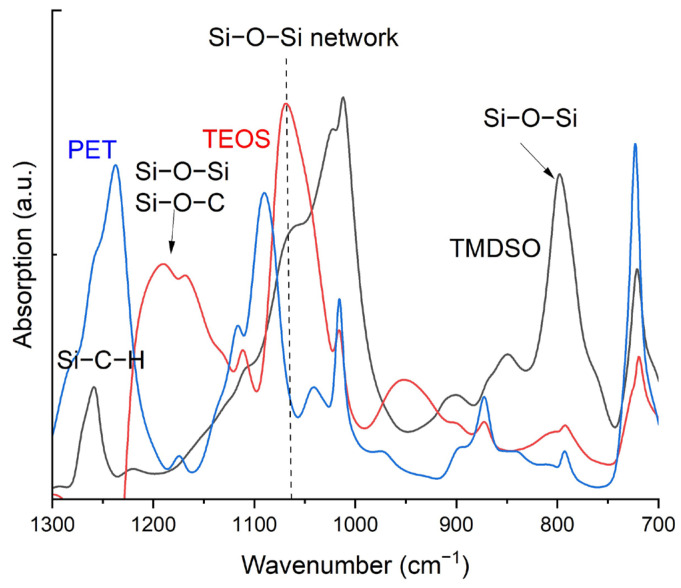
FTIR spectra of the TMDSO (black) and TEOS (red) coatings and uncoated PET (blue).

Figure 8 illustrates FTIR spectra of SiOx coatings using TMDSO and TEOS as well as the uncoated PET. A significant difference in the FTIR spectra of the SiOx coatings from the spectrum of PET was observed. The detailed classification of FTIR on PET films is reported in [46]. Possible assignments presented in the literature are summarized in Table 5. The interpretation of the SiOx FTIR spectra is difficult, and substantial discrepancies in the assignments can be seen in the literature. The FTIR absorptions between 1030 and 1040 cm^−1^ [55,56], between 1060 and 1070 cm^−1^ [25,55,57] and 1110 cm^−1^ [25,55,56] are assigned as the network Si-O structure that is the most important for achieving high gas barrier properties. The regional Si2p XPS analysis indicated that SiOx coating using TEOS contains a much higher content of the SiO_2_ structure. Seeing the intensity of the FTIR absorption, it is likely that the absorption between 1060 and 1070 cm^−1^ is associated with the network Si-O structure. The weak absorption associated with ethylene glycol is also seen in the spectra at 850 and 870 cm^−1^ due to the fact that the absorption from the PET substrate is not perfectly subtracted. In addition to the absorption illustrated in Figure 8, the broad OH absorption at around 3250 cm^−1^ was detected for the SiOx coating using TEOS, associated with its high surface tension. Furthermore, a weak absorption between 2800 and 3000 cm^−1^ was observed, corresponding to the C-H absorption.

### 3.5. OTR and WVTR

Table 6 summarizes the OTR and WVTR results of the PET film and SiOx-coated PET films. OTR at 23 °C, 0% relative humidity (RH) was slightly lowered with the SiOx coating using TMDSO and further decreased with using TEOS. Meanwhile, the WVTR of PET films is significantly affected by moisture at high temperatures. At low-temperature and low-humidity conditions, PET follows the Fickian behavior, in which the relative humidity and the water content of PET have a linear relationship [64]. In other words, the sorption and the diffusion of moisture are balanced in PET. On the other hand, when the temperature and relative humidity are high, the Fickian and swelling phenomena coexist, resulting in high WVTR values. Therefore, if PET films are used under tropical conditions, WVTR measurement at low temperatures or low humidity is insufficient since moisture diffusion mechanisms in PET are different. As with the OTR result, WVTR at 38 °C, 90% RH was slightly lowered with the SiOx coating using TMDSO. However, WVTR was reduced significantly when the SiOx coating using TEOS was deposited. The results support the identification of the structures in the SiOx coatings using FTIR.

Although optimization of the plasma condition should be employed for further improvement of the oxygen and water-vapor barrier properties, the OTR and WVTR results indicate that a large-scale roll-to-roll DBD setup can be used to synthesize SiOx barrier coating.

### 3.6. Industrial Applicability

The present work demonstrated large-scale SiOx synthesis using AP-PACVD, which was not reported in the literature. The detailed experimental conditions and the characterization results presented in Section 2 and Section 3 can be used for practical industrial applications.

## 4. Conclusions

SiOx coating was continuously deposited on PET film by AP-PACVD using a large-scale roll-to-roll device. TMDSO and TEOS were chosen as precursors. XPS measurements indicate that the SiOx coating using TEOS exhibits a higher level of oxidation than that using TMDSO. The regional Si2p XPS suggests that the SiOx coating using TEOS contains SiO_2_ structures associated with a network solid. The characterization of the SiOx coatings using FTIR is not simple, as discrepancies in identification are often seen in the literature. However, the measured FTIR suggests that the network structure, which is important to achieve high barrier properties, of the SiOx coating using TEOS was more pronounced than that using TMDSO. The OTR and WVTR results supported this interpretation.

## Figures and Tables

**Table 1:** Conditions to operate the roll-to-roll AP-PACVD unit to synthesize SiOx coatings. SLM: standard liter per minute.

Precursor		TMDSO	TEOS
Temperature of water bath (°C)		Room temperature	60
Gas flowrate (SLM)	N_2_ (carrier gas)	2.8	20
	N_2_ (dilute gas)	17.2	0
	O_2_	5	5
PET feeding speed (m min^−1^)		4	1
Plasma power (kW)		12	

**Table 2:** Contact angle of deionized water and diiodomethane on the SiOx coatings and the uncoated PET, and their surface-tension values.

Precursor		TMDSO	TEOS	Uncoated
Contact angle (°)	Water	85.0 ± 2.6	17.2 ± 1.2	62.6 ± 2.0
	Diiodomethane	70.1 ± 1.5	44.4 ± 2.0	27.8 ± 1.8
Surface tension (mN/m)	Polar	5.9	35.7	9.3
	Dispersion	22.8	37.3	45.1
	Total	28.7	73.0	54.4

**Table 3:** Elemental composition of SiOx coatings using TMDSO and TEOS.

Precursor		TMDSO	TEOS
Atomic composition (at.%)	Si2p	24.3	24.0
	O1s	54.7	66.5
	C1s	21.0	9.5

**Table 4:** Regional C1s and Si2p XPS results.

	Binding Energy [eV]	TMDSO	TEOS
C1s	285 (C-C, C-H)	94.3%	67.1%
	286.7–287 (C-O)	5.7%	24.8%
	289 (C=O)	0.0%	8.1%
Si2p	102 (Si_2_O or SiO)	57.8%	0.0%
	103.1–103.4 (Si_2_O_3_)	42.2%	44.1%
	104.5 (SiO_2_)	0%	55.9%

**Table 5:** FTIR assignment reported in the literature corresponding to the measured absorptions in Figure 8.

Wavenumber (cm^−1^)	TMDSO	TEOS	Assignment	Ref.
800	✓		Si–O–Si bending	[17,57,58]
			Si–O–Si symmetric stretching	[59]
			Si–C stretching in Si(CH_3_)_2_	[60]
850	✓		CH_2_ stretching in ethylene glycol	[46]
			Si–C stretching in Si(CH_3_)_2_	[60]
			C–H rocking in CH_3_	[61]
870	✓	✓	C–O stretching of ethylene glycol trans-configuration	[46]
900	✓		Si–C stretching in Si(CH_3_)_2_	[60]
950		✓	O–H stretching in Si–OH	[19,55,59]
1030–1040	✓	✓	Si–O–Si linear	[56]
			Si–O–Si asymmetric stretching (network)	[55]
1060–1070	✓	✓	Si–O–Si asymmetric stretching (network)	[25,60]
			Si–O–Si asymmetric stretching (ring)	[55]
1070			Si–O–Si asymmetric stretching	[15,58]
1110	✓	✓	Si–O cage structure	[25]
			open Si–O–C asymmetric stretching	[55]
			Si–O network	[56]
1170		✓	Si–O–Si asymmetric stretching	[16,17,25,59]
1190		✓	Si–O–Si cage structure	[25,52,56]
			C–O–Si stretching	[62]
1260	✓		Si–C–H symmetric bending	[62,63]
			Si–O–C asymmetric stretching	[25]

**Table 6:** OTR at 23 °C, 0% RH, and WVTR at 38 °C, 90% RH. PET film thickness is 12 μm.

	Uncoated	TMDSO	TEOS
OTR (cm^3^ m^−2^ day^−1^)	127.5	114.9	107.3
WVTR (g m^−2^ day^−1^)	53.1	49.4	29.2

## Data Availability

The raw data supporting the conclusions of this article will be made available by the authors upon request.
